# Intensity-modulated radiotherapy alone versus concurrent chemoradiotherapy ± induction chemotherapy in older patients with low-risk stage II or T3N0M0 nasopharyngeal carcinoma

**DOI:** 10.1186/s12885-026-16457-6

**Published:** 2026-07-04

**Authors:** Zhi-Wei Liao, Qizhen Xu, Xue-Song Sun, Chao Lin, Hui-Tang Cai, Liang Hu, Qinghua Wang, Yi-Bin Zhang, Xiao-Long Chen, Li-Jun Zhang, Xiao-Dan Lin, Yun-Hong Tian, Hong-Wei Yu, Yue-Feng Wen

**Affiliations:** 1 Department of Radiation Oncology, Guangzhou Institute of Cancer Research, the Affiliated Cancer Hospital, Guangzhou Medical University, 78 Hengzhigang Road, Guangzhou, 510095 China; 2 Department of Radiation Oncology, Fujian Key Laboratory of Intelligent Imaging and Precision, Clinical Research Center for Radiology and Radiotherapy of Fujian Province, Fujian Medical University Union Hospital, Radiotherapy for Tumors (Fujian Medical University, 29 Xingquan Road, Fuzhou, China; 3 State Key Laboratory of Oncology in South China, Collaborative Innovation Center for Cancer Medicine, Sun Yat-sen University Cancer Center, 651 Dongfeng Road East, Guangzhou, 510060 China; 4 Department of Spinal Surgery, The Sixth Affiliated Hospital of Jinan University (Dongguan East Central Hospital), Dongguan Research Institute for Spinal Cord Injury, 88 Changdong Road, Dongguan, 523500 China; 5 Department of Gastrointestinal Surgery, Guangzhou Institute of Cancer Research, the Affiliated Cancer Hospital, Guangzhou Medical University, 78 Hengzhigang Road, Guangzhou, 510095 China; 6 Department of Radiation Oncology, the Affiliated Cancer Hospital, Guangzhou Institute of Cancer Research, Guangzhou Medical University, 78 Hengzhigang Road, Guangzhou, 510095 China

**Keywords:** Nasopharyngeal carcinoma, Older patients, Low-risk, Intensity-modulated radiotherapy, Chemoradiotherapy, Tumor microenvironment, Hover-Net

## Abstract

**Background:**

The optimal treatment strategy for older patients with low-risk stage II or T3N0M0 nasopharyngeal carcinoma (NPC) remains undefined. This multicenter retrospective study compared the long-term outcomes and toxicities of intensity-modulated radiotherapy (IMRT) alone versus concurrent chemoradiotherapy with or without induction chemotherapy (CCRT ± IC) in this population, and explored the underlying tumor microenvironment (TME) characteristics.

**Methods:**

A total of 1,265 older patients aged ≥ 60 years with low-risk NPC, defined as stage II or T3N0M0 disease without adverse features including lymph node diameter ≥ 3 cm, extranodal extension, or EBV DNA ≥ 4000 copies/mL, were enrolled from three centers between 2010 and 2022. Propensity score matching (PSM) was used to balance baseline characteristics between treatment groups. Survival outcomes and toxicities were compared. Additionally, a deep learning-based Hover-Net model was applied to analyze immune cell infiltration in hematoxylin and eosin-stained sections from a subset of patients to investigate TME differences between risk groups.

**Results:**

After PSM, IMRT alone demonstrated comparable survival outcomes to CCRT ± IC across all cohorts. In the training cohort, the 5-year cancer-specific survival rate was 91.4% in both groups (HR 1.07; *P* = 0.87), with consistent findings for progression-free survival, locoregional relapse-free survival, and distant metastasis-free survival. These results were validated in two external cohorts. However, CCRT ± IC led to significantly higher acute grade 3/4 hematologic and gastrointestinal toxicities. Hover-Net analysis revealed that the low-risk group exhibited significantly higher lymphocyte infiltration in the TME compared with the high-risk group (*P* < 0.05), suggesting a potential biological basis for the favorable outcomes observed with IMRT alone.

**Conclusions:**

For older patients with low-risk stage II or T3N0M0 NPC, IMRT alone achieves survival outcomes equivalent to CCRT ± IC with significantly fewer acute toxicities. The enriched lymphocyte infiltration in the low-risk TME provides a biological rationale for this chemotherapy-sparing approach, supporting IMRT alone as a preferred toxicity-de-escalation strategy.

**Supplementary Information:**

The online version contains supplementary material available at https://doi.org/10.1186/s12885-026-16457-6.

## Background

Nasopharyngeal carcinoma (NPC) is a malignant tumor with a distinct geographical distribution, exhibiting high incidence rates in Southern China and Southeast Asia [1]. Pathologically, NPC is predominantly characterized by undifferentiated, non-keratinizing squamous cell carcinoma, which is highly sensitive to radiotherapy (RT), thus establishing RT as the cornerstone of treatment [2, 3].

Concurrent chemoradiotherapy (CCRT) has been recommended as the standard treatment for stage II NPC based on a prospective clinical trial that compared CCRT with conventional RT alone in stage II NPC patients and showed significant survival benefits from CCRT in the two-dimensional conventional radiotherapy era [4]. However, over the past two decades, with the widespread application of intensity-modulated radiotherapy (IMRT), the locoregional control rate of NPC has significantly increased, while the survival benefit from chemotherapy has noticeably decreased. Therefore, the benefit of chemotherapy additional to IMRT for stage II NPC remains controversial.

Individualized stratified treatment based on risk stratification factors such as tumor size, extranodal extension, and pretreatment Epstein–Barr virus (EBV) DNA level is proposed in the current National Comprehensive Cancer Network (NCCN) guidelines (version 2; 2025) to better manage patients with stage II NPC. Recently, the ninth version of the AJCC/UICC nasopharyngeal cancer TNM staging classification has incorporated refined anatomical criteria to improve prognostic stratification and support more risk-adapted therapeutic decision-making [5]. Concurrently, the landscape of systemic therapy for NPC has evolved substantially, with increasing emphasis on biomarker-guided and individualized chemotherapy or immunotherapy strategies based on clinical and biological risk factors [6]. The NCCN guidelines recommend IMRT alone for low-risk stage II or T3N0M0 NPC patients without adverse features, based on a prospective clinical trial [7], which defined low-risk as T3N0 or T1N1 disease with a single unilateral lymph node ≤ 3 cm, no level IV/Vb nodes, and pretreatment EBV DNA < 4000 copies/mL. However, this clinical trial excluded patients older than 65 years due to poorer performance status, age-related comorbidities, and diminished organ function, resulting in a current lack of evidence-based treatment guidelines for this subgroup.

Therefore, we conducted this retrospective, Southern China data-based, multicenter, real-world study to compare the efficacy and toxicity between IMRT alone and CCRT with or without induction chemotherapy (CCRT ± IC) in older patients with low-risk stage II or T3N0M0 NPC. Notably, while the definition of “older patients” in oncology continues to evolve, we adopted a threshold of ≥ 60 years, which is based on its established use in NPC clinical studies and the earlier age of onset characteristic of endemic regions in Southern China; importantly, this chronological age cutoff was complemented by geriatric-related assessment metrics, including the ACE-27 comorbidity index and Karnofsky Performance Status score, to better reflect physiological vulnerability and treatment tolerance.

## Materials and methods

### Patients

This multicenter retrospective study enrolled 1,265 newly diagnosed older patients with NPC treated between January 2010 and December 2022 at the Affiliated Cancer Hospital, Guangzhou Medical University (training cohort), Sun Yat-sen University Cancer Center (validation cohort 1), and Fujian Medical University Union Hospital (validation cohort 2). The inclusion criteria were defined as follows: (a) age ≥ 60 years; (b) histologically confirmed WHO type II or III nasopharyngeal squamous cell carcinoma; (c) stage II or T3N0M0 according to the 8th American Joint Committee on Cancer/Union for International Cancer Control (AJCC/UICC) staging system, specifically T1N1M0, T2N0M0, T2N1M0, or T3N0M0; (d) no distant metastasis at initial diagnosis; (e) no history of secondary primary malignancies; (f) absence of high-risk features, including lymph node necrosis, extranodal extension, lymph node maximum diameter ≥ 3 cm, or EBV DNA ≥ 4000 copies/mL; (g) received radical IMRT-based treatment, including IMRT alone or cisplatin-based CCRT with or without platinum-based induction chemotherapy; (h) Karnofsky Performance Status (KPS) score ≥ 70, with no severe underlying heart, lung, liver, or kidney disease; and (i) availability of complete medical records and follow-up data.

The study protocol was approved by the Institutional Review Boards or Ethics Committees of all participating centers. Given the retrospective design, the requirement for individual patient informed consent was waived.

### Treatment protocol

All patients underwent radical IMRT, with treatment planning and delivery adhering to the guidelines of the International Commission on Radiation Units and Measurements Report 83. Prescribed doses were as follows: gross tumor volume of the nasopharynx (GTVnx), 66–70 Gy; gross tumor volume of involved lymph nodes (GTVnd), 64–68 Gy; high-risk clinical target volume (CTV1), 60–64 Gy; and low-risk clinical target volume (CTV2), 50–54 Gy. A conventional fractionation schedule was employed, with 1.8–2.2 Gy per fraction administered once daily, five times per week, over a total course of 6.5-7 weeks.

The CCRT regimen consisted of cisplatin-based concurrent chemotherapy, primarily as monotherapy, administered as either cisplatin 75 mg/m² every 3 weeks or weekly cisplatin 30–40 mg/m². Patients in the IC + CCRT group received platinum-based doublet induction chemotherapy, such as docetaxel/cisplatin, gemcitabine/cisplatin, or paclitaxel/cisplatin, prior to CCRT. Typically, 2–3 cycles were administered, with one cycle every 3 weeks. Doses were adjusted based on patient age, performance status, and renal function. The Adult Comorbidity Evaluation-27 (ACE-27) was used to assess comorbidities [8].

### Follow-up and toxicity assessment

Patients underwent systematic follow-up every 3 months for the first 2 years, and then annually until death, the most recent follow-up, or the occurrence of an endpoint event. Routine follow-up comprised detailed medical history, head and neck physical examination, fiberoptic nasopharyngoscopy, enhanced MRI of the nasopharynx and neck, chest CT, abdominal ultrasound or CT, routine blood tests, biochemical tests, and quantitative plasma EBV DNA. Plasma EBV DNA was quantified using a real-time quantitative PCR assay targeting the BamHI-W fragment of the EBV genome, a method consistently performed across all three centers throughout the study period. Supplementary examinations, such as PET-CT or bone scans, were conducted selectively based on clinical suspicion or imaging abnormalities.

Follow-up duration was calculated from the first day of treatment to the occurrence of an endpoint event or the last follow-up. Acute adverse events during treatment were documented and graded according to the National Cancer Institute Common Terminology Criteria for Adverse Events version 5.0. Chronic or late radiation toxicities after treatment were evaluated using the Radiation Therapy Oncology Group/European Organisation for Research and Treatment of Cancer late radiation morbidity scoring schema.

### Pathological image processing and cell typing

To investigate differences in immune cell infiltration within the TME between different risk groups, pathological sections from 77 patients with NPC at the Affiliated Cancer Hospital of Guangzhou Medical University were collected. The cohort was divided into a low-risk group (38 cases) and a high-risk group (39 cases) according to the risk model established in this study. All pathological sections were stained with hematoxylin and eosin and digitally scanned using a 3DHISTECH PANNORAMIC SCAN II whole-slide scanner to obtain high-resolution digital pathological images.

This study employed the deep learning-based Hover-Net model for automatic nuclear segmentation and classification within digital pathological images. Hover-Net is an advanced algorithm that achieves precise nuclear instance segmentation by integrating three parallel branches: a nuclear classification branch, a pixel prediction branch, and a distance map prediction branch [9]. In the nuclear segmentation stage, the pixel prediction branch was used to determine whether each pixel belonged to a nuclear region, thereby distinguishing nuclei from background tissue. The distance map prediction branch generated distance maps by calculating horizontal and vertical distances from pixels within a nucleus to the center of their respective nuclear clusters, enabling precise separation of adjacent or overlapping nuclei. Based on Hover-Net’s preset classifier, four key cell types within the TME were identified and quantified: epithelial cells, lymphocytes, macrophages, and neutrophils. Statistical analysis was then performed on the densities of these infiltrating cells between different risk groups.

### Study endpoints and statistical analysis

Continuous variables including age, BMI, and EBV DNA were converted into categorical variables using predefined clinical thresholds: age < 65 versus ≥ 65 years, BMI < 18.5 versus ≥ 18.5 kg/m², and EBV DNA < 1000 versus ≥ 1000 copies/mL. For EBV DNA, the cut-off value of 1000 copies/mL was selected based on its established prognostic significance in NPC [10] and to achieve balanced distribution across treatment groups in propensity score matching. Categorical variables were compared between the IMRT group and CCRT ± IC group using the chi-square test or Fisher’s exact test, as appropriate.

A 1:1 nearest-neighbor propensity score matching method was applied to address baseline differences between the IMRT group and CCRT ± IC group in each cohort. Caliper values were 0.05 for the training cohort, 0.06 for validation cohort 1, and 0.05 for validation cohort 2. Matching variables included age, sex, histological type, T stage, N stage, TNM stage, lymph node maximum axial diameter, EBV DNA, ACE-27 score, smoking history, KPS score, and BMI. Matching quality was evaluated using standardized mean differences, with SMD < 10% indicating adequate balance.

The primary endpoint was cancer-specific survival (CSS). Secondary endpoints included progression-free survival (PFS), locoregional relapse-free survival (LRFS), and distant metastasis-free survival (DMFS). Survival rates were estimated using the Kaplan-Meier method, with intergroup differences assessed using the log-rank test. Cox proportional hazards models were used to analyze treatment-by-covariate interactions. Covariates included age, sex, T stage, N stage, TNM stage, and EBV DNA. Multivariate analysis was also performed using the Cox proportional hazards model. Statistical significance was set at α = 0.05, two-sided. Statistical analyses were conducted using SPSS 27.0 and R version 4.4.2.

## Results

### Patient characteristics

**Fig. 1:**
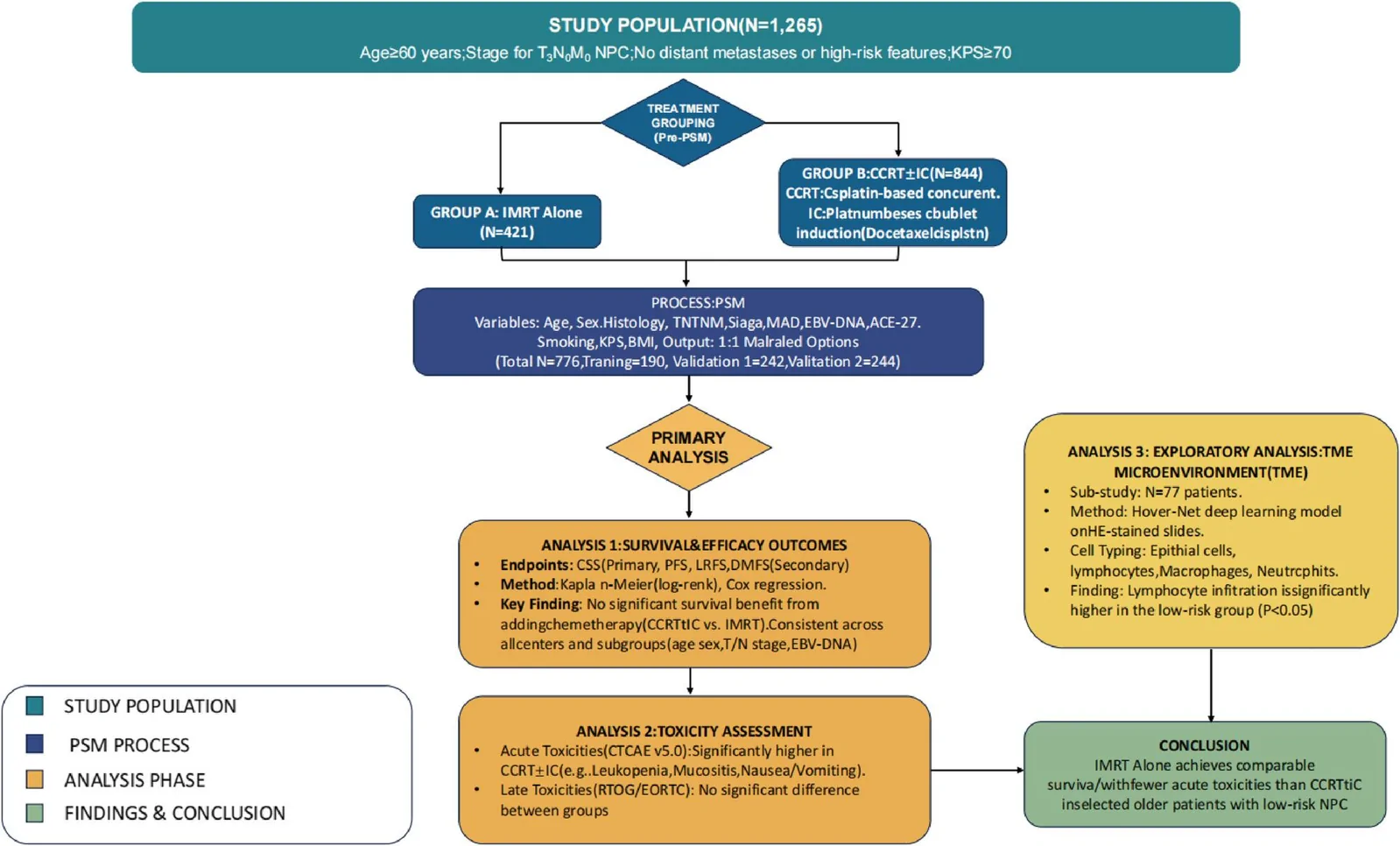
The Study Enrollment Flowchart

There were 418, 461, and 386 older patients with low-risk stage II or T3N0M0 NPC included in the training cohort and two external validation cohorts, respectively. The median follow-up time was 60.0 months (IQR 40.0–86.2) for the training cohort, 60.2 months (IQR 38.4–89.6) for validation cohort 1, and 61.5 months (IQR 40.1–91.0) for validation cohort 2. Treatment modalities were categorized as either IMRT alone or CCRT ± IC, with respective ratios of 102:316, 189:272, and 130:256 in the training cohort and validation cohorts, respectively. The study enrollment and patient selection process are shown in Fig. 1. After PSM, baseline characteristics between the two groups were comparable, with 190, 342, and 244 matched patients included in the training cohort, validation cohort 1, and validation cohort 2, respectively.(*P* > 0.05, Table 1).

**Table 1:** Baseline clinical characteristics of patients from three cohorts after matching

Characteristics	Training cohort (*n* = 190)			Validation cohort 1 (*n* = 342)			Validation cohort 2 (*n* = 244)		
	IMRT	CCRT ± IC	*P* value	IMRT	CCRT ± IC	*P* value	IMRT	CCRT ± IC	*P* value
Total	95	95		171	171		122	122	
Age, years, *n* (%)			0.77			0.5			0.79
< 65	37 (38.9)	35 (36.8)		107 (62.6)	113 (66.1)		80 (65.6)	82 (67.2)	
≥ 65	58 (61.1)	60 (63.2)		64 (37.4)	58 (33.9)		42 (34.4)	40 (32.8)	
Sex, *n* (%)			1.00			0.63			0.66
Female	29 (30.5)	29 (30.5)		52 (30.4)	48 (28.1)		33 (27.0)	30 (24.6)	
Male	66 (69.5)	66 (69.5)		119 (69.6)	123 (71.9)		89 (73.0)	92 (75.4)	
Histology, *n* (%)			1.00			0.31			0.39
WHO type II	10 (10.5)	10 (10.5)		6 (3.5)	10 (5.8)		8 (6.6)	5 (4.1)	
WHO type III	85 (89.5)	85 (89.5)		165 (96.5)	161 (94.2)		114 (93.4)	117 (95.9)	
T stage^a^, *n* (%)			0.90			0.51			0.37
T1	10 (10.5)	10 (10.5)		23 (13.5)	22 (12.9)		21 (17.2)	30 (24.6)	
T2	73 (76.8)	75 (79.0)		95 (55.6)	86 (50.3)		74 (60.7)	68 (55.7)	
T3	12 (12.6)	10 (10.5)		53 (30.9)	63 (36.8)		27 (22.1)	24 (19.7)	
N stage^a^, *n *(%)			0.62			0.28			0.20
N0	26 (27.4)	23 (24.2)		96 (56.1)	86 (50.3)		62 (50.8)	52 (42.6)	
N1	69 (72.6)	72 (75.8)		75 (43.9)	85 (49.7)		60 (49.2)	70 (57.4)	
TNM stage^a^, *n* (%)			0.65			0.25			0.64
II	83 (87.4)	85 (89.5)		118 (69.0)	108 (63.2)		95 (77.9)	98 (80.3)	
III	12 (12.6)	10 (10.5)		53 (31.0)	63 (36.8)		27 (22.1)	24 (19.7)	
MAD, cm, *n* (%)			0.53			0.69			0.29
< 2	83 (87.4)	80 (84.2)		156 (91.2)	158 (92.4)		106 (86.9)	100 (82.0)	
≥ 2	12 (12.6)	15 (15.8)		15 (8.8)	13 (7.6)		16 (13.1)	22 (18.0)	
EBV DNA^b^, copies/mL, *n* (%)			0.11			0.9			0.35
< 1000	84 (88.4)	76 (80.0)		129 (75.4)	130 (76.0)		99 (81.1)	93 (76.2)	
≥ 1000	11 (11.6)	19 (20.0)		42 (24.6)	41 (24.0)		23 (18.9)	29 (23.8)	
ACE-27 scores, *n* (%)			0.10			0.78			0.55
< 2	94 (98.9)	90 (94.7)		164 (95.9)	165 (96.5)		117 (95.9)	115 (94.3)	
≥ 2	1 (1.1)	5 (5.3)		7 (4.1)	6 (3.5)		5 (4.1)	7 (5.7)	
Smoking, *n* (%)			0.37			0.06			0.44
No	63 (66.3)	57 (60.0)		106 (62.0)	89 (52.0)		63 (51.6)	57 (46.7)	
Yes	32 (33.7)	38 (40.0)		65 (38.0)	82 (48.0)		59 (48.4)	65 (53.3)	
KPS, *n* (%)			0.76			0.7			0.73
70–80	89 (93.7)	90 (94.7)		167 (97.7)	168 (98.2)		118 (96.7)	117 (95.9)	
90–100	6 (6.3)	5 (5.3)		4 (2.3)	3 (1.8)		4 (3.3)	5 (4.1)	
BMI, kg/m^2^, *n* (%)			0.25			0.43			1.00
< 18.5	13 (13.7)	8 (8.4)		9 (5.3)	6 (3.5)		6 (4.9)	6 (4.9)	
≥ 18.5	82 (86.3)	87 (91.6)		162 (94.7)	165 (96.5)		116 (95.1)	116 (95.1)	

*Abbreviations* *IMRT*   intensity-modulated radiotherapy, *CCRT  * concurrent chemoradiotherapy, *IC  * induction chemotherapy, *MAD  * Maximal axial diameter, *EBV  * Epstein–Barr virus, *ACE  * Adult Comorbidity Evaluation, *KPS  * Karnofsky performance scales, *BMI  * Body Mass Index

a: According to the 8th edition of AJCC/UICC Staging System. b: All variables were measured before treatment

### Chemotherapy adherence and dose intensity

In the CCRT ± IC group, the median number of completed concurrent cisplatin cycles was 2 (IQR 2–3) across all cohorts. Specifically, 68.4% (65/95), 71.3% (122/171), and 69.7% (85/122) of patients in the training cohort, validation cohort 1, and validation cohort 2, respectively, completed at least 2 cycles of concurrent cisplatin chemotherapy. The mean cumulative cisplatin dose was 171.2 ± 31.6 mg/m². Among patients who received induction chemotherapy (*n* = 31, 55, and 42 in the three cohorts, respectively), the median number of completed induction cycles was 2 (IQR 2–3), with 83.9%, 80.0%, and 81.0% completing the planned 2–3 cycles. These data indicate that chemotherapy adherence in this older population was moderate, and suboptimal dose intensity in some patients may partially explain the lack of additional survival benefit observed with CCRT ± IC compared to IMRT alone.

### Survival outcomes

**Fig. 2:**
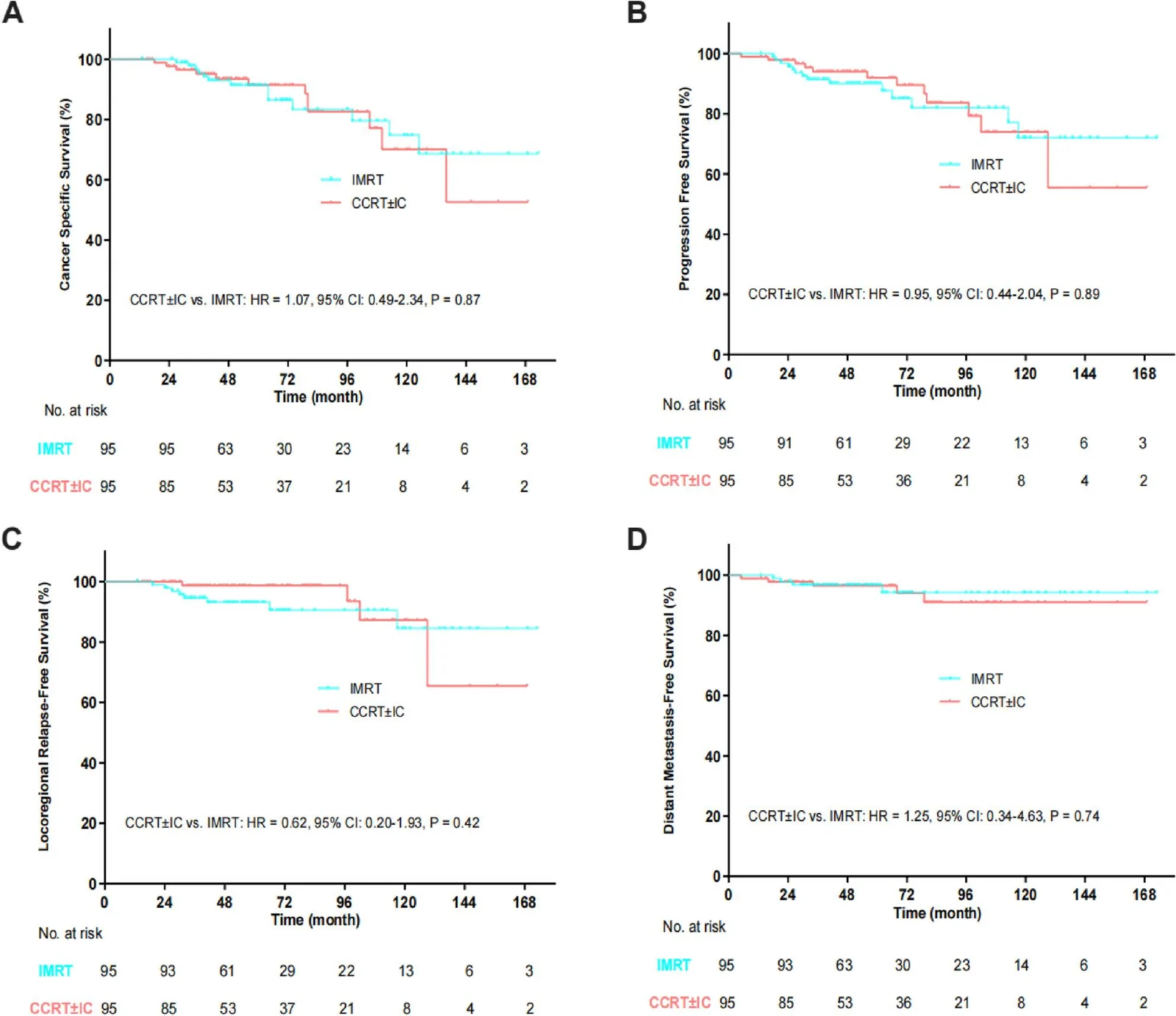
Kaplan-Meier survival curve estimates for the IMRT group versus the CCRT±IC group after matching at Affiliated Cancer Hospital & Institute of Guangzhou Medical University, showing cancer-specific survival (CSS) (**A**), progression-free survival (PFS) (**B**), locoregional relapse-free survival (LRFS) (**C**), and distant metastasis-free survival (DMFS) (**D**). P-values were calculated using the unadjusted log-rank test. IMRT: intensity-modulated radiotherapy; CCRT: concurrent chemoradiotherapy; IC: induction chemotherapy; HR: hazard ratio; CI: confidence interval

In the training cohort, the 5-year CSS rates were 91.4% for both the CCRT ± IC and IMRT groups (HR 1.07, 95% CI: 0.49–2.34, *P* = 0.87, Fig. 2A). The 5-year PFS rates were 91.9% versus 90.1% (HR 0.95, 95% CI: 0.44–2.04, *P* = 0.89, Fig. 2B), LRFS rates were 98.7% versus 93.2% (HR 0.62, 95% CI: 0.20–1.93, *P* = 0.42, Fig. 2C), and DMFS rates were 96.5% versus 96.8% (HR 1.25, 95% CI: 0.34–4.63, *P* = 0.74, Fig. 2D) for the CCRT ± IC and IMRT groups, respectively, with no statistically significant differences observed.

**Fig. 3:**
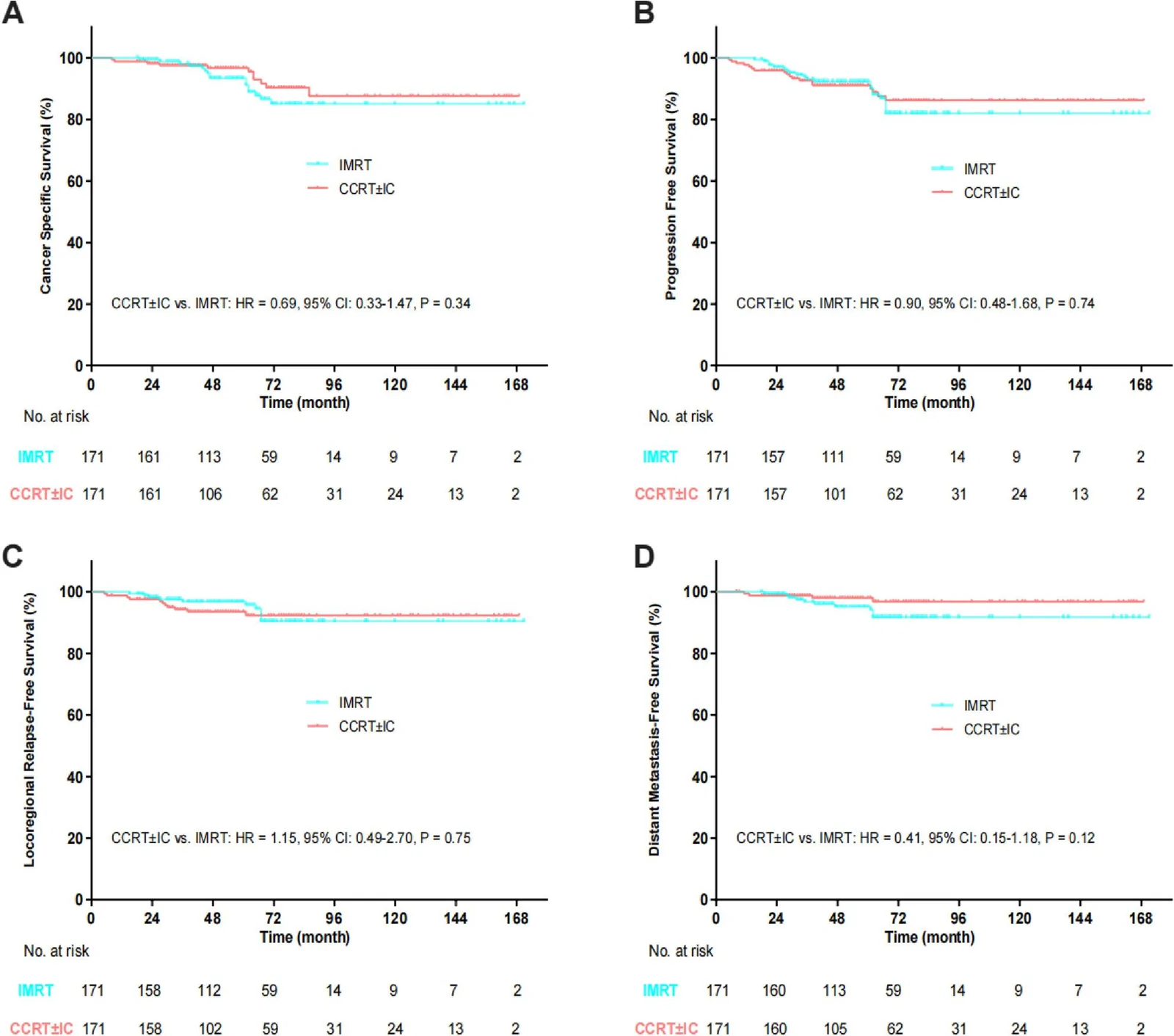
Kaplan-Meier survival curve estimates for the IMRT group versus the CCRT±IC group after matching at Sun Yat-sen University Cancer Center, showing cancer-specific survival (CSS) (**A**), progression-free survival (PFS) (**B**), locoregional relapse-free survival (LRFS) (**C**), and distant metastasis-free survival (DMFS) (**D**). *P*-values were calculated using the unadjusted log-rank test. IMRT: intensity-modulated radiotherapy; CCRT: concurrent chemoradiotherapy; IC: induction chemotherapy; HR: hazard ratio; CI: confidence interval

**Fig. 4:**
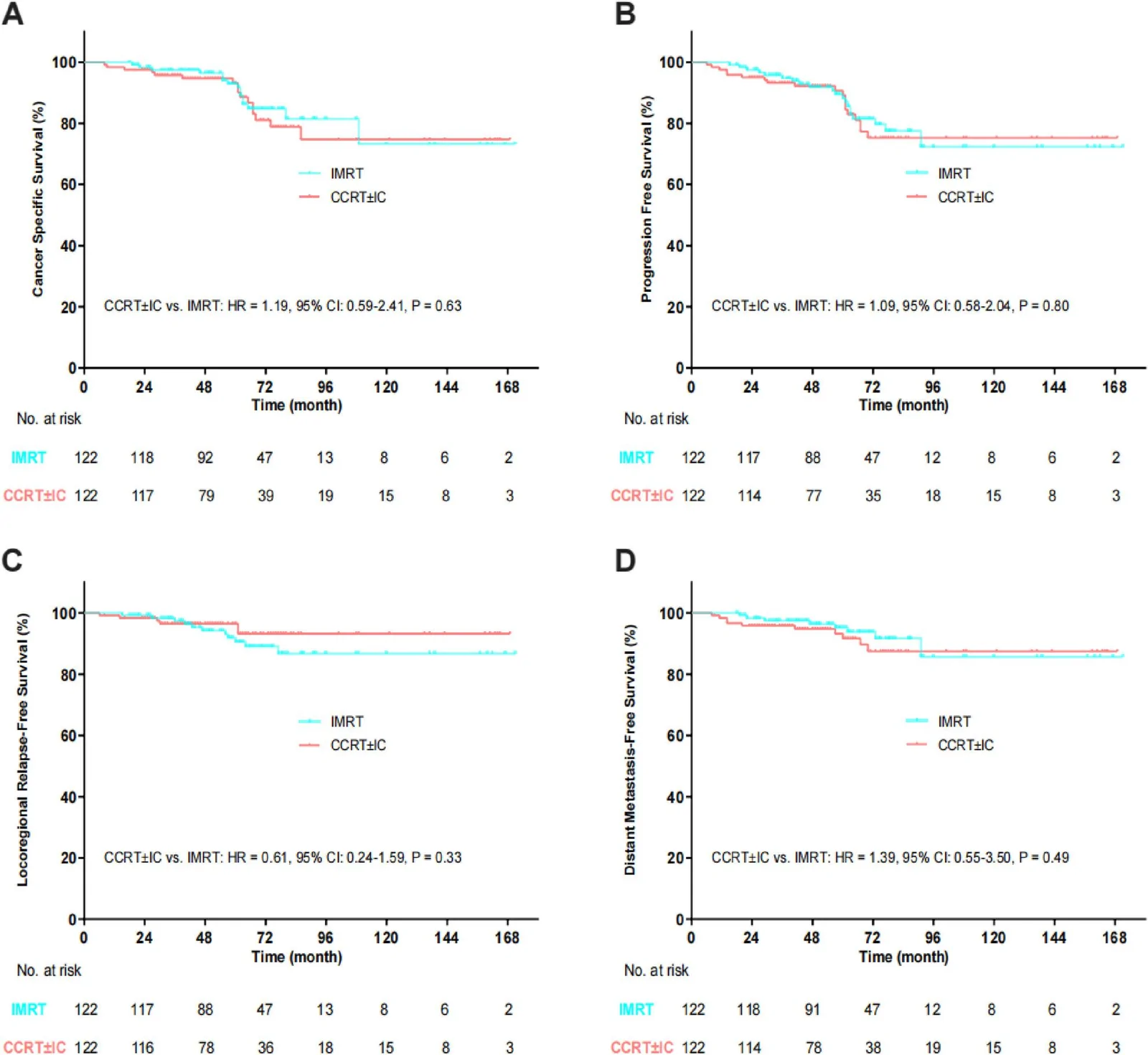
Kaplan-Meier survival curve estimates for the IMRT group versus the CCRT±IC group after matching at Fujian Medical University Union Hospital, showing cancer-specific survival (CSS) (**A**), progression-free survival (PFS) (**B**), locoregional relapse-free survival (LRFS) (**C**), and distant metastasis-free survival (DMFS) (**D**). P-values were calculated using the unadjusted log-rank test. IMRT: intensity-modulated radiotherapy; CCRT: concurrent chemoradiotherapy; IC: induction chemotherapy; HR: hazard ratio; CI: confidence interval

These results were further validated in the two validation cohorts. In validation cohort 1, the 5-year CSS, PFS, LRFS, and DMFS rates were comparable between the CCRT ± IC group and IMRT group (CSS: 96.7% vs. 93.3%, *P* = 0.34; PFS: 91.1% vs. 92.3%, *P* = 0.74; LRFS: 93.5% vs. 96.8%, *P* = 0.75; DMFS: 98.0% vs. 95.2%, *P* = 0.12, Fig. 3A-D). In validation cohort 2, the 5-year CSS, PFS, LRFS, and DMFS rates were also comparable between the CCRT ± IC group and IMRT group (CSS: 93.2% vs. 93.0%, *P* = 0.63; PFS: 89.2% vs. 88.2%, *P* = 0.80; LRFS: 96.5% vs. 90.6%, *P* = 0.33; DMFS: 91.7% vs. 95.2%, *P* = 0.49, Fig. 4A-D). Multicenter data consistently indicated that CCRT ± IC did not confer additional survival benefits compared to IMRT alone.

### Patterns of disease failure

Up to the last follow-up, in the training cohort, the locoregional relapse rates in the IMRT group and CCRT ± IC group were 8.4% and 4.2%, respectively; distant metastasis rates were 4.2% and 5.3%; disease progression rates were 14.7% and 12.6%; and cancer-related mortality rates were 13.7% and 12.6%. Similar treatment outcomes were also observed in the validation cohorts, further confirming no statistically significant differences in recurrence, metastasis, disease progression, or mortality between the two treatment groups (*P* > 0.05, shown in Table 2).

**Table 2:** Distribution of disease failure

Event	Training cohort (*n* = 190)			Validation cohort 1 (*n* = 342)			Validation cohort 2 (*n* = 244)		
	IMRT (*n* = 95)	CCRT ± IC (*n* = 95)	*P* value	IMRT (*n* = 171)	CCRT ± IC (*n* = 171)	*P* value	IMRT (*n* = 122)	CCRT ± IC (*n* = 122)	*P* value
Locoregional relapse, *n* (%)	8 (8.4)	4 (4.2)	0.23	10 (5.8)	11 (6.4)	0.82	11 (9.0)	6 (4.9)	0.21
Distant metastasis, *n* (%)	4 (4.2)	5 (5.3)	0.73	10 (5.8)	4 (2.3)	0.10	8 (6.6)	10 (8.2)	0.62
Progression, *n* (%)	14 (14.7)	12 (12.6)	0.67	21 (12.3)	18 (10.5)	0.61	20 (16.4)	19 (15.6)	0.86
Cancer-specific death, *n* (%)	13 (13.7)	12 (12.6)	0.83	16 (9.4)	11 (6.4)	0.32	15 (12.3)	16 (13.1)	0.85

*Abbreviations* *IMRT  * intensity-modulated radiotherapy, *CCRT  * concurrent chemoradiotherapy, *IC  * induction chemotherapy

### Subgroup analyses

**Fig. 5:**
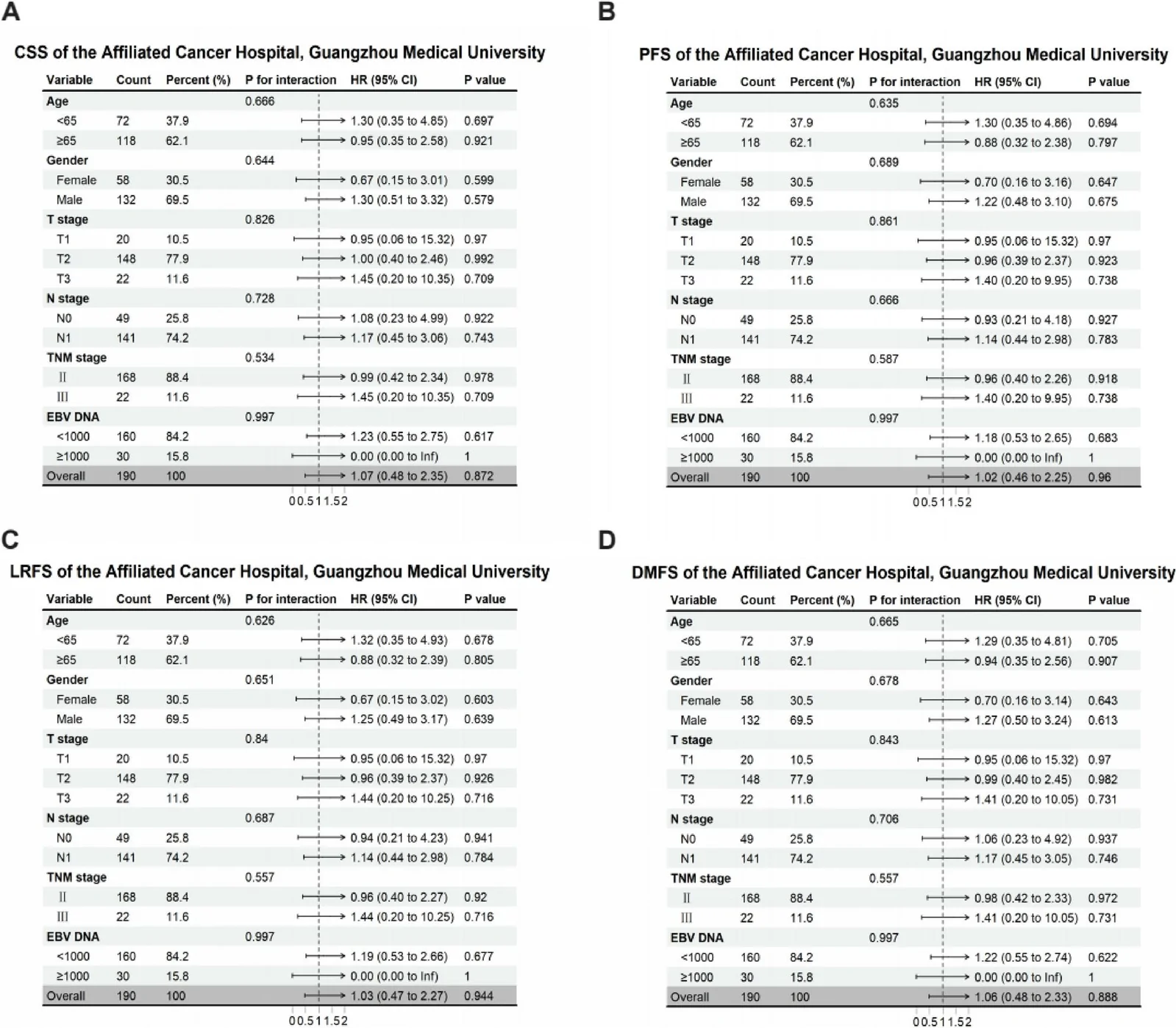
Subgroup survival analyses for the IMRT group versus the CCRT±IC group at Affiliated Cancer Hospital & Institute of Guangzhou Medical University, showing cancer-specific survival (CSS) (**A**), progression-free survival (PFS) (**B**), locoregional relapse-free survival (LRFS) (**C**), and distant metastasis-free survival (DMFS) (**D**). IMRT: intensity-modulated radiotherapy; CCRT: concurrent chemoradiotherapy; IC: induction chemotherapy; HR: hazard ratio; CI: confidence interval

In the predefined subgroup analyses according to patients’ baseline characteristics, including age, sex, T stage, N stage, TNM stage, and plasma EBV DNA levels, no significant differences in CSS were observed between the IMRT group and CCRT ± IC group in the training cohort (Fig. 5A). Further analysis also demonstrated no statistically significant differences in PFS (Fig. 5B), LRFS (Fig. 5C), or DMFS (Fig. 5D) between the two groups across all predefined subgroups. Moreover, consistent results were observed in the two validation cohorts (Figures S1–S2).

### Toxicity profiles

Integrated toxicity data from the three participating centers showed that CCRT ± IC was associated with a higher burden of acute treatment-related toxicities than IMRT alone, whereas late toxicities were generally comparable between the two groups. In the training cohort, the CCRT ± IC group had significantly higher incidences of grade 3/4 leukopenia (10.5% vs. 1.1%, *P* = 0.02) and neutropenia (8.4% vs. 1.1%, *P* = 0.01) than the IMRT group. Similar trends were observed in the validation cohorts, particularly for leukopenia and neutropenia.

For non-hematologic acute toxicities, patients receiving CCRT ± IC experienced more frequent gastrointestinal reactions, including nausea and vomiting, as well as a higher incidence of mucositis compared with those receiving IMRT alone. Hepatotoxicity was also more common in the CCRT ± IC group, although most events were grade 1/2 and grade 3/4 hepatotoxicity was rare. In contrast, nephrotoxicity showed no statistically significant difference between the two treatment groups across the cohorts.

Late toxicities, including xerostomia, neck fibrosis or injury, trismus, and hearing impairment, were mostly grade 1/2 and did not differ significantly between the IMRT and CCRT ± IC groups. The detailed distributions of acute and late toxicities across all cohorts are presented in Table 3.Overall, these findings indicate that the addition of chemotherapy increased acute hematologic and gastrointestinal toxicities but did not substantially increase late radiation-related toxicities.

**Table 3:** Acute and Late Adverse Events Related to Radiotherapy and Chemotherapy

Event		Training cohort (*n* = 190)			Validation cohort 1 (*n* = 342)			Validation cohort2(*n* = 244)			
		IMRT	CCRT ± IC	*P* value	IMRT	CCRT ± IC	*P* value	IMRT		CCRT ± IC	*P* value
Acute toxicities, *n* (%)											
Leukopenia				0.02			<0.01				0.02
G0		38 (40.0)	30 (31.6)		62 (36.2)	46 (26.9)		47 (38.5)		42 (34.4)	
G1/2		56 (58.9)	55 (57.9)		106 (62.0)	107 (62.6)		73 (59.8)		68 (55.7)	
G3/4		1 (1.1)	10 (10.5)		3 (1.8)	18 (10.5)		2 (1.6)		12 (9.8)	
Neutropenia				0.01^*^			<0.01				0.52^*^
G0		41 (43.2)	27 (28.4)		62 (36.2)	40 (23.4)		39 (32.0)		36 (29.5)	
G1/2		53 (55.8)	60 (63.2)		106 (62.0)	119 (69.6)		81 (66.4)		81 (66.4)	
G3/4		1 (1.1)	8 (8.4)		3 (1.8)	12 (7.0)		2 (1.6)		5 (4.1)	
Anaemia				0.38^*^			0.42				0.75^*^
G0		35 (36.8)	28 (29.5)		72 (42.1)	65 (38.0)		43 (35.2)		37 (30.3)	
G1/2		56 (58.9)	65 (68.4)		95 (55.6)	98 (57.3)		75 (61.5)		80 (65.6)	
G3/4		4 (4.2)	2 (2.1)		4 (2.3)	8 (4.7)		4 (3.3)		5 (4.1)	
Thrombocytopenia				0.59^*^			0.70^*^				0.83^*^
G0		88 (92.6)	86 (90.5)		153 (89.5)	149 (87.1)		111 (91.0)		108 (88.5)	
G1/2		6 (6.3)	9 (9.5)		17 (9.9)	20 (11.7)		10 (8.2)		13 (10.7)	
G3/4		1 (1.1)	0 (0.0)		1 (0.6)	2 (1.2)		1 (0.8)		1 (0.8)	
Hepatoxicity				<0.01^*^			0.02^*^				<0.01^*^
G0		92 (96.8)	77 (81.1)		153 (89.5)	137 (80.1)		114 (93.4)		98 (80.3)	
G1/2		3 (3.2)	17 (17.9)		18 (10.5)	33 (19.3)		8 (6.6)		23 (18.9)	
G3/4		0 (0.0)	1 (1.1)		0 (0.0)	1 (0.6)		0 (0.0)		1 (0.8)	
Nephrotoxicity				0.14^*^			0.42^*^				0.17^*^
G0		84 (88.4)	76 (80.0)		154 (90.1)	150 (87.7)		109 (89.3)		101 (82.8)	
G1/2		11 (11.6)	17 (17.9)		17 (9.9)	19 (11.1)		13 (10.7)		19 (15.6)	
G3/4		0 (0.0)	2 (2.1)		0 (0.0)	2 (1.2)		0 (0.0)		2 (1.6)	
Nausea				<0.01			<0.01				<0.01
G0		83 (87.4)	41 (43.2)		155 (90.6)	91 (53.2)		110 (90.2)		68 (55.7)	
G1/2		10 (10.5)	40 (42.1)		14 (8.2)	58 (33.9)		10 (8.2)		40 (32.8)	
G3/4		2 (2.1)	14 (14.7)		2 (1.2)	22 (12.9)		2 (1.6)		14 (11.5)	
Vomiting				<0.01			<0.01				<0.01
G0		83 (87.4)	37 (38.9)		155 (90.6)	97 (56.7)		110 (90.2)		64 (52.5)	
G1/2		12 (12.6)	34 (35.8)		14 (8.2)	48 (28.1)		12 (9.8)		34 (27.9)	
G3/4		0 (0.0)	24 (25.3)		2 (1.2)	26 (15.2)		0 (0.0)		24 (19.7)	
Mucositis				<0.01			0.04				0.02
G0		28 (29.5)	10 (10.5)		34 (19.9)	24 (14.0)		34 (27.9)		18 (14.8)	
G1/2		60 (63.2)	70 (73.7)		120 (70.2)	115 (67.3)		78 (63.9)		86 (70.4)	
G3/4		7 (7.4)	15 (15.8)		17 (9.9)	32 (18.7)		10 (8.2)		18 (14.8)	
Dermatitis				0.16^*^			<0.01				0.09^*^
G0		83 (87.4)	75 (78.9)		139 (81.3)	115 (67.3)		110 (90.2)		107 (87.7)	
G1/2		11 (11.6)	15 (15.8)		32 (18.7)	56 (32.7)		12 (9.8)		10 (8.2)	
G3/4		1 (1.1)	5 (5.3)		0 (0.0)	0 (0.0)		0 (0.0)		5 (4.1)	
Late toxicities, *n* (%)											
Xerostomia				0.76^*^			0.74^*^				0.90^*^
G0		32 (33.7)	34 (35.8)		77 (45.0)	73 (42.7)		49 (40.2)		47 (38.5)	
G1/2		63 (66.3)	60 (63.2)		94 (55.0)	97 (56.7)		73 (59.8)		74 (60.7)	
G3/4		0 (0.0)	1 (1.1)		0 (0.0)	1 (0.6)		0 (0.0)		1 (0.8)	
Neck damage				0.44^*^			0.11^*^				0.22^*^
G0		71 (74.7)	63 (66.3)		133 (77.8)	116 (67.8)		94 (77.0)		82 (67.2)	
G1/2		23 (24.2)	31 (32.6)		36 (21.1)	51 (29.8)		27 (22.1)		37 (30.3)	
G3/4		1 (1.1)	1 (1.1)		2 (1.2)	4 (2.3)		1 (0.8)		3 (2.5)	
Trismus				1.00^*^			1.00^*^				
G0		92 (96.8)	91 (95.8)		168 (98.2)	167 (97.7)		119 (97.5)		118 (96.7)	1.00^*^
G1/2		3 (3.2)	4 (4.2)		3 (1.8)	4 (2.3)		3 (2.5)		4 (3.3)	
G3/4		0 (0.0)	0 (0.0)		0 (0.0)	0 (0.0)		0 (0.0)		0 (0.0)	
Hearing impairment				0.61^*^			0.34^*^				0.07^*^
G0		49 (51.6)	42 (44.2)		101 (59.1)	88 (51.5)		68 (55.7)		50 (41.0)	
G1/2		44 (46.3)	50 (52.6)		68 (39.8)	80 (46.8)		52 (42.6)		69 (56.6)	
G3/4		2 (2.1)	3 (3.2)		2 (1.2)	3 (1.8)		2 (1.6)		3 (2.5)	

*Abbreviations* *IMRT *intensity-modulated radiationtherapy, *CCRT * concurrent chemoradiotherapy, *IC * induction chemotherapy

*These items were calculated with Fisher’s exact test

### Multivariate analysis

Multivariate analysis showed that, in the training cohort, after adjusting for confounding factors including age, sex, T stage, N stage, TNM stage, lymph node maximum axial diameter, EBV DNA, and ACE-27 score, treatment protocol did not emerge as a significant prognostic factor for CSS (HR 0.68, 95% CI 0.35–1.30, *P* = 0.24), PFS (HR 0.78, 95% CI 0.43–1.40, *P* = 0.40), LRFS (HR 0.60, 95% CI 0.27–1.32, *P* = 0.20), or DMFS (HR 0.83, 95% CI 0.29–2.34, *P* = 0.72). These findings were further confirmed in the validation cohorts.Further details of the multivariate analysis are shown in Table 4, Table S1, Table S2.

**Table 4:** Multivariable analysis of prognostic factors in the training cohort

Characteristics	HR	95% CI	*P* value
Cancer specific survival			
Age, years (≥ 65 vs.<65)	1.09	0.59–2.01	0.80
Sex (male vs. female)	1.57	0.73–3.34	0.25
T stage (T3 vs. T2 vs. T1)	1.68	0.50–5.65	0.40
N stage (N1 vs. N0)	0.72	0.28–1.84	0.49
TNM stage (III vs. II)	0.90	0.17–4.68	0.90
MAD, cm (≥ 2 vs.<2)	2.07	1.0-4.28	0.05
EBV DNA, copies/mL (≥ 1000 vs.<1000)	1.34	0.50–3.58	0.56
ACE-27 scores (≥ 2 vs.<2)	1.82	0.54–6.10	0.33
Treatment (CCRT ± IC vs. IMRT)	0.68	0.35–1.30	0.24
Progression free survival			
Age, years (≥ 65 vs.<65)	1.05	0.61–1.81	0.85
Sex (male vs. female)	2.19	1.06–4.52	0.04
T stage (T3 vs. T2 vs. T1)	0.98	0.41–2.36	0.97
N stage (N1 vs. N0)	0.95	0.41–2.21	0.90
TNM stage (III vs. II)	1.41	0.37–5.44	0.62
MAD, cm (≥ 2 vs.<2)	1.40	0.72–2.71	0.32
EBV DNA, copies/mL (≥ 1000 vs.<1000)	1.47	0.67–3.22	0.34
ACE-27 scores (≥ 2 vs.<2)	1.42	0.43–4.65	0.57
Treatment (CCRT ± IC vs. IMRT)	0.78	0.43–1.40	0.40
Locoregional relapse-free survival			
Age, years (≥ 65 vs.<65)	1.29	0.59–2.83	0.53
Sex (male vs. female)	2.67	0.90–7.90	0.08
T stage (T3 vs. T2 vs. T1)	0.76	0.22–2.67	0.67
N stage (N1 vs. N0)	0.53	0.18–1.57	0.25
TNM stage (III vs. II)	1.70	0.28–10.21	0.56
MAD, cm (≥ 2 vs.<2)	1.27	0.47–3.43	0.63
EBV DNA, copies/mL (≥ 1000 vs.<1000)	2.80	1.05–7.47	0.04
ACE-27 scores (≥ 2 vs.<2)	2.30	0.52–10.22	0.28
Treatment (CCRT ± IC vs. IMRT)	0.60	0.27–1.32	0.20
Distant metastasis-free survival			
Age, years (≥ 65 vs.<65)	0.96	0.39–2.38	0.93
Sex (male vs. female)	3.37	0.77–14.69	0.11
T stage (T3 vs. T2 vs. T1)	1.27	0.29–5.66	0.75
N stage (N1 vs. N0)	NC	NC	0.95
TNM stage (III vs. II)	NC	NC	0.95
MAD, cm (≥ 2 vs.<2)	1.65	0.62–4.38	0.31
EBV DNA, copies/mL (≥ 1000 vs.<1000)	1.14	0.32–4.02	0.84
ACE-27 scores (≥ 2 vs.<2)	2.00	0.45–8.92	0.37
Treatment (CCRT ± IC vs. IMRT)	0.83	0.29–2.34	0.72

*Abbreviations IMRT  * intensity-modulated radiationtherapy, *CCRT  * concurrent chemoradiotherapy, *IC  * induction chemotherapy, *MAD  * Maximal axial diameter, *EBV  * Epstein–Barr virus, *ACE  * Adult Comorbidity Evaluation. NC HR could not be calculated as no incident occurred in the N0 stage or II stage subgroup

### Tumor microenvironment analysis

To provide a mechanistic basis for our clinical observation that low-risk patients achieved favorable outcomes with IMRT alone, we further investigated the distribution characteristics of four key cell types (epithelial cells, lymphocytes, macrophages, and neutrophils) within the tumor microenvironment (TME) between high-risk and low-risk patients. This analysis aimed to test the hypothesis that a favorable immune milieu may underlie the effectiveness of a chemotherapy-sparing strategy, thereby transforming an empirical clinical finding into a biologically rationalized therapeutic approach.

**Fig. 6:**
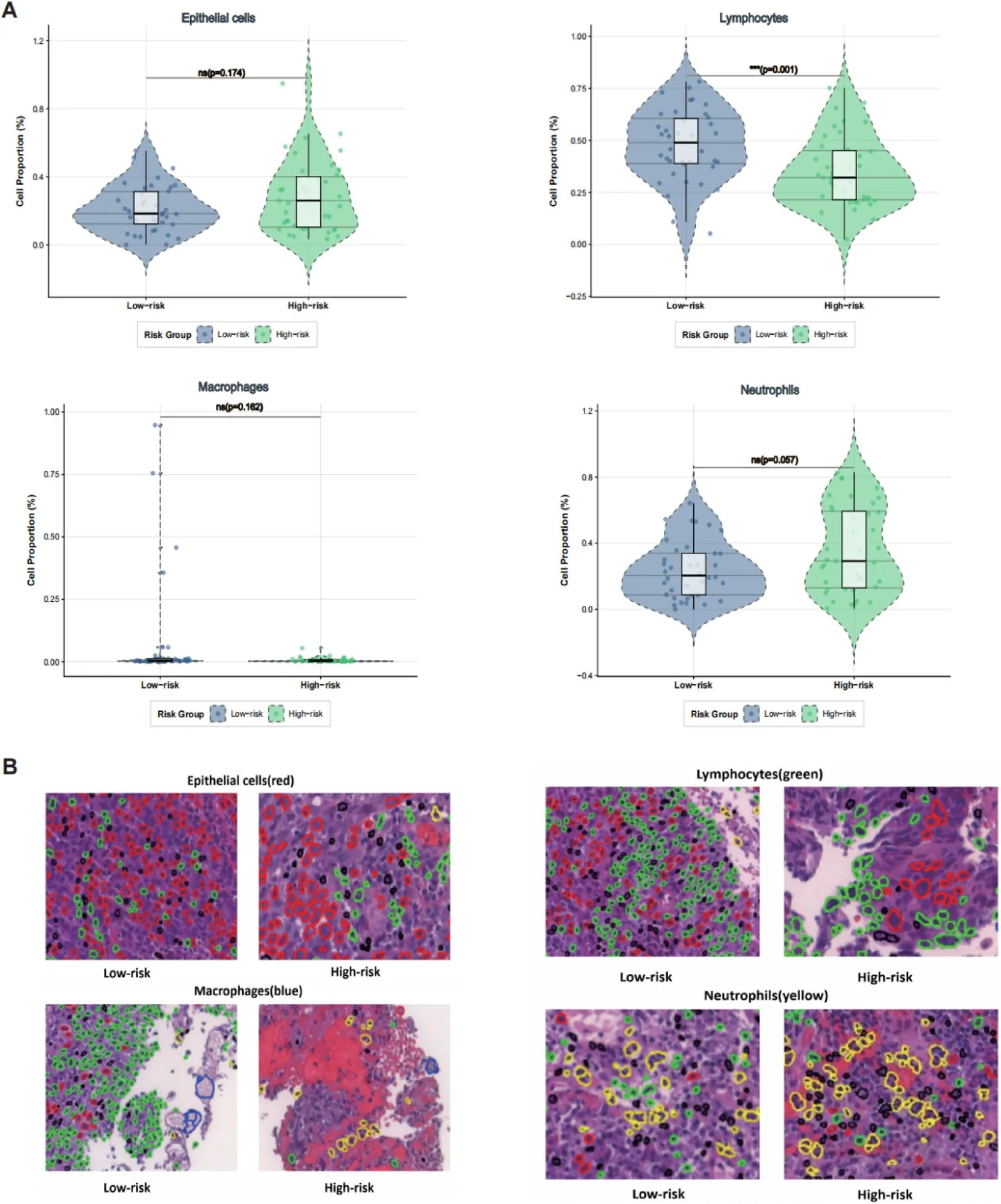
**A** Violin plots show the infiltration of four major cell types across risk groups. Lymphocyte infiltration was significantly higher in the low-risk group (*P* < 0.05), indicating a favorable prognosis. No significant differences were observed for epithelial cells, macrophages, or neutrophils, although neutrophils tended to be higher in the high-risk group (*P* > 0.05). Between-group comparisons were performed using the Wilcoxon rank-sum test. **B **Representative cell segmentation images display spatial distribution differences of the four cell types between low- and high-risk patients. Labels and colors below each image mark the key cell types (e.g., red circles for epithelial cells). This color-coding enables direct comparison of spatial distribution and infiltration density between groups

Risk stratification analysis based on the prognostic model revealed significant heterogeneity in cellular infiltration patterns across different risk groups (Fig. 6A). Specifically, no significant differences were observed in the abundance of epithelial cells or macrophages between the high-risk and low-risk groups, suggesting that the quantities of these two cell types may not be directly associated with risk stratification. Notably, lymphocyte infiltration levels demonstrated a significant correlation with risk grouping. The analysis showed that lymphocyte infiltration in the tumor microenvironment of low-risk patients was significantly higher than that in high-risk patients, and this difference was statistically significant (*P* < 0.05), indicating that lymphocyte enrichment may be closely associated with more favorable prognosis and low-risk characteristics. Regarding neutrophils, although a trend toward increased infiltration abundance was observed in the high-risk group, this difference did not reach statistical significance (*P* > 0.05), and its potential role warrants further investigation. Representative cell segmentation images visually illustrated the distribution differences of the four cell types between risk groups (Fig. 6B). Consistent with the quantitative analysis results, significantly higher lymphocyte density was observed in tumor tissues of low-risk patients compared to high-risk patients, further supporting the value of lymphocyte infiltration levels as a potential indicator for risk stratification.

## Discussion

Older patients are usually excluded from prospective clinical studies due to diminished organ reserve and poorer treatment tolerance, leading to an evidence gap for this specific population [11, 12]. Therefore, directly applying guidelines developed primarily for younger or general adult populations to older patients is questionable. The benefit of chemotherapy combined with IMRT for older patients with NPC remains controversial, especially for those with stage II or T3N0M0 disease without adverse features. To the best of our knowledge, this is the first retrospective study conducted at three cancer centers in Southern China to investigate long-term outcomes and toxicities of CCRT ± IC compared with IMRT alone in older patients with low-risk stage II or T3N0M0 NPC without adverse features.

Our findings indicated that for older patients with low-risk stage II or T3N0M0 NPC, IMRT alone achieved comparable long-term survival outcomes and treatment failure patterns to CCRT ± IC. However, CCRT ± IC was associated with a significantly increased incidence and severity of acute toxicities compared with IMRT alone. Moreover, prespecified subgroup analyses based on baseline characteristics demonstrated no significant differences in survival outcomes across different age, sex, T/N stage, or EBV DNA subgroups. By incorporating EBV DNA levels, ACE-27 comorbidity index, and KPS functional status score, and by employing multicenter PSM to control for confounding factors, our findings provide rational evidence supporting a chemotherapy de-escalation strategy for older patients with low-risk stage II or T3N0M0 NPC.

Several previous studies have assessed the efficacy and toxicity of chemoradiotherapy in older patients with NPC, with results partially aligning with our findings. Chen et al. reported that in older patients aged ≥ 65 years with locoregionally advanced NPC, induction plus concurrent chemoradiotherapy versus concurrent chemoradiotherapy did not significantly improve survival outcomes but markedly increased acute toxicities, such as grade ≥ 3 leukopenia and mucositis [12]. Li et al. used recursive partitioning analysis to stratify older NPC patients into different risk groups and suggested that only middle- or high-risk groups might benefit from chemotherapy, whereas IMRT alone may be optimal for the low-risk group [13]. Jin et al. demonstrated that no survival benefit from additional neoadjuvant chemotherapy was observed in low-risk older patients with stage III NPC [14]. Wu et al. found that among low-risk older NPC patients defined by EBV DNA and T stage, those treated with IMRT alone had comparable survival rates to those treated with chemoradiotherapy [15]. He et al. demonstrated that the low-risk subgroup of older patients identified by a prognostic nomogram did not gain survival benefit from IC + CCRT compared with CCRT [16]. However, our previous study found that CCRT significantly improved survival compared with IMRT alone in older patients with stage II NPC [17]. The discrepancy may be explained by the fact that the previous study did not perform detailed risk stratification and included patients both with and without adverse features, potentially introducing heterogeneity in current clinical practice.

Stage II NPC is a population with considerable prognostic heterogeneity [18]. Several studies have revealed significant associations between MRI-related lymph node features, such as cervical nodal necrosis, extracapsular spread, and nodal size, and prognosis in stage II NPC [19–21]. Thus, it is imperative to incorporate lymph node features and pretreatment EBV DNA level as risk stratification factors for older patients with stage II NPC. In the present study, the low-risk criteria proposed for older patients with stage II or T3N0M0 NPC, including no nodal necrosis or extracapsular extension, lymph node size < 3 cm, and EBV DNA < 4000 copies/mL, provided a clinically practical foundation for risk stratification. Further refinement may enhance clinical applicability. Incorporating dynamic circulating tumor DNA monitoring may improve prognostic accuracy [22]. In addition, although ACE-27 score and age may partially reflect treatment tolerance, formal geriatric assessment that systematically evaluates functional status, comorbidities, cognition, nutrition, and social support may more effectively identify patients at high risk for treatment-related toxicities [8, 23]. Furthermore, artificial intelligence and deep learning-based models integrating radiological, clinical, and molecular data may help refine precise risk stratification and identify optimal candidates for RT alone [24, 25].

Owing to incomplete organ reserve function and a high burden of comorbidities, older patients exhibit significantly reduced tolerance to chemotherapy, potentially leading to treatment-related toxicities that offset survival benefits [26]. In our study, significantly higher incidence and severity of acute toxicities, including leukopenia, neutropenia, mucositis, nausea, vomiting, and hepatotoxicity, were observed in the chemoradiotherapy group. Previous studies also revealed increased treatment-related toxicities in older patients, consistent with our results [12, 17]. These findings suggest that chemotherapy de-escalation strategies offer important advantages in reducing treatment-related toxicities, which is particularly crucial for older patients.

Plasma EBV DNA level has been identified as a robust prognostic marker for NPC patients [10]. Additionally, several previous studies demonstrated that older age and lymph node maximum axial diameter were associated with poorer prognosis in NPC patients [27–30]. Our present study also confirmed the prognostic relevance of EBV DNA, age, and nodal size, further emphasizing the importance of incorporating these factors into risk stratification for older patients with stage II NPC.

We further investigated TME characteristics across risk groups. This study employed the deep learning-based Hover-Net model for automatic nuclear segmentation and classification within digital pathological images. Using this model, we identified and quantified four key cell types within the TME: epithelial cells, lymphocytes, macrophages, and neutrophils.

Lymphocyte infiltration was significantly higher in the low-risk group. As tumor-infiltrating lymphocytes are key effectors of adaptive immunity and established prognostic markers in solid tumors, this suggests that enhanced endogenous antitumor immune surveillance may underlie the favorable outcomes observed in low-risk patients. This finding is consistent with previous reports indicating that lymphocyte infiltration may serve as a predictor of radiotherapy benefit [31–33]. No significant differences were found in epithelial cell or macrophage counts between groups, implying that absolute cell numbers alone may not determine prognosis. Functional polarization, such as M1/M2 macrophage status, may be more clinically relevant [34, 35]. Neutrophils trended higher in the high-risk group but without statistical significance, possibly due to sample size limitations and phenotypic plasticity, warranting further study [36]. Enhanced lymphocyte infiltration in low-risk patients may provide a biological basis for effective IMRT alone, as endogenous immunity can potentiate radiotherapy-related immune effects and sustain long-term tumor control without chemotherapy. This immune-clinical correlation supports treatment de-escalation and suggests that assessing TME immune status may help identify patients likely to maintain efficacy without chemotherapy-associated toxicity.

In real-world clinical practice, the decision to proceed with IMRT alone versus CCRT ± IC was influenced by several factors. Physicians at the participating centers generally referenced the recommendations of the NCCN guidelines, but the final treatment decision was individualized based on a comprehensive evaluation of patient age, performance status, comorbidity burden assessed by the ACE-27 score, pretreatment EBV DNA level, nodal characteristics, and patient preference after multidisciplinary discussion. Patients perceived to have poorer tolerance or higher risk of chemotherapy-related toxicities were more likely to receive IMRT alone. Although we employed rigorous PSM to balance measured confounders, we cannot exclude the possibility of residual selection bias due to unmeasured factors such as frailty, nutritional status, or social support. This inherent limitation of retrospective studies should be considered when interpreting the comparable survival outcomes between the two groups, and prospective randomized trials are warranted to confirm our findings.

In summary, our results suggest that for older NPC patients with stage II or T3N0M0 disease, IMRT alone represents a reasonable treatment strategy after precise screening of the low-risk population. These findings highlight the importance of geriatric-adapted treatment personalization and support tailored therapeutic strategies that account for age-specific physiological vulnerabilities, thereby achieving an optimal balance between oncological control and treatment tolerability.

Nevertheless, the present study has several limitations. First, because this was a retrospective real-world analysis, it may be susceptible to selection bias. Although baseline characteristics were well balanced through PSM, the results may still be influenced by unmeasured confounding factors, such as nutritional status, social support, treatment compliance, and frailty. Additionally, the lack of data on quality of life, cognitive function, financial toxicity, and dynamic EBV DNA monitoring limited comprehensive assessment of long-term benefit-risk profiles. Furthermore, dynamic monitoring of EBV DNA levels was not standardized across centers, and variations in detection assays and uncertainty regarding cut-off values may introduce inconsistency. Moreover, our study excluded patients with KPS < 70, who constitute a particularly vulnerable subgroup of older NPC patients with substantial unmet clinical needs. The optimal management for these patients, including the role of radiotherapy alone, palliative care, or best supportive care, remains undefined and warrants dedicated prospective investigation in frail older populations. Future research should incorporate patient-reported outcome questionnaires, longitudinal plasma EBV DNA monitoring, and standardized geriatric assessment to systematically evaluate the overall benefits in terms of efficacy, toxicity, and quality of life.

## Conclusions

In this retrospective multicenter study, for older patients with low-risk stage II or T3N0M0 NPC, IMRT alone achieved survival outcomes comparable to those of CCRT ± IC while significantly reducing acute toxicities. Enriched lymphocyte infiltration in the low-risk TME may provide a biological rationale for this chemotherapy-sparing approach. These findings support IMRT alone as a preferred toxicity-de-escalation strategy for carefully selected older patients with low-risk NPC.

## Supplementary Information

Supplementary Material 1.

Supplementary Material 2.

## Data Availability

The datasets generated and/or analyzed during the current study are available from the corresponding author on reasonable request.

## Abbreviations

IMRT: Intensity-modulated radiotherapy
CCRT: Concurrent chemoradiotherapy
IC: Induction chemotherapy
NPC: Nasopharyngeal carcinoma
PSM: Propensity score matching
CSS: Cancer-specific survival
PFS: Progression-free survival
LRFS: Locoregional relapse-free survival
DMFS: Distant metastasis-free survival
EBV: Epstein-Barr virus
MAD: Maximal axial diameter
ACE-27: Adult comorbidity evaluation-27
KPS: Karnofsky performance status
BMI: Body mass index
HE: Hematoxylin and eosin
NC: Not calculable
TME: Tumor microenvironment
GTV: Gross tumor volume
CTV: Clinical target volume
RTOG: Radiation therapy oncology group
EORTC: European organisation for research and treatment of cancer
CTCAE: Common terminology criteria for adverse events
AJCC: American joint committee on cancer
UICC: Union for international cancer control
